# Supplementary material for: Clinical epidemiological characteristics and antibiotic sensitivity of Escherichia coli urinary tract infection
Source: PLoS One. 2025 Nov 11;20(11):e0336572. doi: 10.1371/journal.pone.0336572 (PMC12604755; doi:10.1371/journal.pone.0336572)
Supplement: Attachment — Submitted filename: Reviewers comments .docx [file pone.0336572.s004.docx]

Clinical epidemiological characteristics and drug sensitivity characteristics of urinary tract infection of Escherichia coli

Submission: Major revision

The authors of this paper have identified an opportunity to correlate retrospective hospital case data, samples in a hospital biobank with hospitalization records for urinary tract infection as a result of *Escherichia coli*. The authors intended approach to associate the antibiotic susceptibility profiles for the *E.coli* strains and their corresponding Extended-Spectrum β-lactamase status with hospitalization cases and perhaps source of infection is a unique perspective, which if properly articulated can allow hospital systems develop protocols to reviewing antibiotic use or discontinued use within hospital systems.

The authors have however not clearly articulated their thoughts in the current report. The paper will benefit from a major revision across all sections of the paper from abstract to discussion. In the current state, the paper is hard to follow, requiring major editorial input. Some quick but not exhaustive list of highlights below.

- There are some vague terminologies used in the paper which, if clearly defined would greatly assist the readers is understanding the flow of thought presented here.
- Inconsistencies in the use of word between in-hospital, out of hospital etc>>. This needs to be properly defined and consistent in presentation across all the sections of the paper as it is crucial in the results interpretation. What does out of hospital infection mean? Infections obtained outside the hospital whilst in-hospital refers to infections obtained in the hospital? In this case a strong association can be determined between ESBL status and infection acquisition which would be a relevant finding.
- Was bacterial culture on fresh urine samples undertaken in this study or was analysis on retrospective stored bacterial isolates done? Authors are advised to report the precise methods employed in this study.
- How did the authors arrive at sensitivity values for each antibiotic type? Can this be highlighted under the methods section?
- Under statistical methods, the authors refer to metrological data- please clarify how this relates to the current study!!
- In many cases they repeatedly used Escherichia coli when they could have simply written E.coli.
- Also p value is written with the small letter p and not capitalized within text.
- ESBL status relative to days of hospitalization was highlighted in table 2 what bearing does this have on the patient statuses i.e. of out-of hospital and in-hospital that come across strongly in this paper. What is the relationship between ESBL and these two definitions? Is that what was intended to be captured in Table 1?
- Under the General situation of research subjects in the results section this statement reads **‘**A total of 401 **study subjects** that met the **inclusion criteria** were included in this study- there was never a place preceding or after this section where the precise inclusion criteria were clearly highlighted, whilst this may be assumed, it would be clearer to the readers if this can be properly defined. Also under the header titled ‘In-hospital infection and out-of-hospital infection’ the statement refers **‘**A total of 401 **patients** were included in this study- should this not rather read as samples from 401 patients or study subjects? Also be consistent for ease of readability. This section will be a good place to further reiterate the definition of in-hospital infection vs out-of-hospital infection. Authors are advised to rewrite the entire section to ensure clarity of message.
