# Supplementary material for: Clinical epidemiological characteristics and antibiotic sensitivity of Escherichia coli urinary tract infection
Source: PLoS One. 2025 Nov 11;20(11):e0336572. doi: 10.1371/journal.pone.0336572 (PMC12604755; doi:10.1371/journal.pone.0336572)
Supplement: Attachment — Submitted filename: rebuttal-PONE YC.docx [file pone.0336572.s006.docx]

**Point-by-point response to Referees’ comments**

**Reviewer #1:**

INTRODUCTION
The introduction is logical and flows well. The prevalence/epidemiological context are clearly stated. Also, the clinical relevance is well established. However, the followings should be clarified.
1.Clarity. “Under certain circumstances”. Specify some of the circumstances e.g., immunosuppression or catheterization.

**Response**: Thank you for highlighting the need for specificity. We have revised the phrase 'under certain circumstances' to explicitly state relevant clinical contexts. The text now reads: “such as window infections caused by diarrhea or dysentery, immunosuppression or catheterization”. (line 46-47)

2. Quantitative specification. “UTI is considered the second common cause of infectious diseases” …. Reference and state specific epidemiological data or global incidence rankings to support this claim.

**Response**: Thank you for requesting epidemiological clarification. We have revised the statement to reflect authoritative sources and added quantitative context: following respiratory tract infections, and account for more than seven million office visits and more than one million visits to emergency departments in the United States, necessitating 100,000 hospitalizations” with new references. (line 52-54)
3. UTI classification. … “UTI is classified as a complex and non-complex” … Add a brief definition of what constitutes a complex UTI for clarification to the readers.

**Response**: Thank you for this constructive suggestion. We have corrected the word "recurrent" to "complex". (line 60)

3. Terminology precision. “Parenteral pathogenic” is a bit confusing. Change this to “extra intestinal pathogenic” if referring to UPEC’s ability to cause infections outside the gut.

**Response**: Thank you for this important clarification. We agree that 'parenteral pathogenic' may cause confusion in this context. The text has been revised in the revised manuscript. (line 64)

5. Closing statement. … “to reveal their drug resistance and clinical epidemiological characteristics”. This can be ended with a stronger and precision phase such as “with the goal of guiding effective antimicrobial treatment plan and public health intervention”.

**Response**: Thank you for your suggestion. We all agreed that your advice was very professional,and we have revised it in the revised manuscript. (line 70)

6. … “admitted from January 2023 to December 2023” …. Specify if this is a multicenter study or single hospital dataset.

**Response**: Thank you for highlighting this important detail. We confirmed that this is a single-center study. The revised text now explicitly states: The clinical data of 401 cases of urinary culture *E. coli* who were treated with duplicate strains at Xuancheng City People's Hospital from January 2023 to December 2023 (repeated strains have been removed) (line 67-68)

METHODS
Ethics
1. Break into clearer sentences

**Response**: Thank you for your reminder, we have revised the relevant sentences.
2. Ensure formal tone and compliance with international ethics reporting standards.
For example, “This study included Escherichia coli isolates cultured from urine samples of hospitalized patients between January 1, 2023, and December 31, 2023. Ethical approval was obtained from the Ethics Committee of Xuancheng City People's Hospital (Approval Number: 2022-pjky005-01). Written informed consent was not required as the study used bacterial isolates collected as part of routine clinical care, and all patient data were anonymized and de-identified prior to analysis”.

**Response**: Thank you for emphasizing ethical reporting rigor. We have revised it in the revised manuscript. (line 73-78)

Experimental procedure
1. Improve on the grammar and flow.

**Response**: Thanks for your reminder. We have improved the grammar of the manuscript.
2. Change typographical error “form” to “from” (patients form 01/01/2023 to 31/12/2023).
**Response**: Thank you for noting this oversight. The error has been corrected. (line 83)
Bacterial culture
The opening sentence is unclear. Remove or rewrite if it must be there. Streamline method steps for clarity.
**Response**: Thank you for your reminder. The opening sentence has been removed in the revised manuscript.

4. STATISTICAL ANALYSIS
   1. Correct grammar and punctuations

**Response:** We have improved the grammar and punctuation in the revised manuscript.
2. Ensure tense consistency (past tense).

**Response:** We have modified the tense of the sentence in the article to keep it consistent.
3. Clarify statistical methods and match with international reporting standards.

**Response:** Thanks for your professional advice. We have matched the statistical methods used in this study and international reporting standards. (line 107-111)
4. Ensure consistency in units (e.g. μL)

**Response**: We have made changes in the revised manuscript. (line 97)

5. Spacing and software naming

**Response**: We have adjusted the spacing to make sure the correct software name was used. (line111)

RESULTS
General situation of research subjects
1. Clarity and formality
1. Use precise and formal phrasing e.g. subjects to patients/cases.Statistical test to statistical analysis.

**Response**: Thank you for your professional advice. We have modified it in the revised manuscript based on your suggestions. (line 114)
2. Avoid redundancy e.g. “statistical results show….. which is statistically significant is repetitive.

**Response**: Thank you for highlighting this redundancy. We have made changes in the revised manuscript. (line 117-119)
2. Numerical reporting
1. Report p-values consistently with standard formatting (e.g. P = 0.252, do not write in italics).

**Response**: Thank you for emphasizing this important formatting detail. We confirm all p-values are now reported consistently throughout the revised manuscript. (line 119, 123, 183, etc.)
2. Remove parentheses around mean ± SD values except required.

**Response**: Thank you for your reminder. We have removed parentheses at this position according to your suggestion. (line 116, 117, 120, and 121)
3. Terminology consistency
I would suggest using hospital-acquired and non-hospital acquired infections instead of “in-hospital” and “out-of-hospital” for standardization.

**Response**: Thank you for your suggestion. We have replaced "hospital-acquired" with "in-hospital" and “non-hospital acquire” with “out-of-hospital” in the whole revised manuscript. (line 26, 27, 29, 56, and 131,etc)
4. Grammar and style
1. Improve sentence flow and eliminate awkward phrasing.

**Response**: Thanks for your reminder. We have revised the sentences in the article to ensure their fluency.
2. Clarify confusing logic. E.g. P>0.05 was significant. This contradicts standard significance thresholds. In other words, this does not signify significance, yet the statement claims the opposite. Confirm the intended meaning and adjust accordingly.

**Response**: Thank you for your reminder. We found that this is a typo, and we have changed "p>0.05" to "P<0.001" in the revised manuscript. (line 151)
Drug susceptibility
1. Manufacturer name error. “Merrier” should be corrected to bioMérieux.

**Response**: Thanks for your reminder. We have revised it. (line 156)
2. Redundancy and flow. The phrase ‘Escherichia coli exhibited high sensitivity to. Most antibiotics with rates exceeding 80% and some surpassing 90%...” is a bit repetitive. Group antibiotics into classes for better readability.

**Response**: Thank you for this constructive suggestion. We have revised the text to: The *E. coli* isolates exhibited notable susceptibility (>80%) to a wide range of antibiotic classes, such as aminoglycosides, cephalosporins, and carbapenems. Particularly, imipenem (IPM) and ertapenem (ETP) displayed remarkable efficacy. (line 157-159)

3.Scientific tone
1. Change drug sensitivity to antimicrobial susceptibility for precision.

**Response**: Thank you. We have systematically replaced “drug sensitivity” with “antimicrobial susceptibility” throughout the manuscript to align with standard clinical microbiology terminology (CLSI M100 guidelines). (line 153)
2. State clearly which findings require clinical caution e.g. low fluoroquinolone sensitivity.

**Response**: Thank you for this constructive suggestion. In the last sentence of the chapter describing each antibiotic sensitivity, we have revised it to remind clinical quinolones that have the lowest sensitivity. (line 165-166)
3. Ensure that drug abbreviations (e.g., AMK, ETP) are defined.

**Response**: Thank you. We have standardized several antibiotic abbreviations based on the latest CLSI M100 guidelines. For example, cefuroxime sodium (CXM), imipenem (IPM), etc. (line 159, 161, 165, etc)
4. Highlight any multidrug resistance patterns briefly.

**Response**: Thank you for your professional advice. We have briefly introduced CRE in the revised manuscript. (line 167-171)

DISCUSSION
1. Grammar and language use

**Response**: Thank you for your careful review and valuable suggestions. We have revised the sentences in the article to ensure their fluency.
1. Rewrite the multiple redundant constructions

**Response**: Thank you for reminding us that we have appropriately deleted the redundant content in the "discussion" section.
2. Inconsistent verb tenses (e.g. it was found that… to is...)

**Response**: We have revised the text to ensure all descriptions of our study’s results consistently use past tense (e.g., changing 'is' to 'was' in line 190).
3. Change this phrase … “bacteria detection time to e.g. time to detection” for clarity.

**Response**: Thank you for your insightful comment. We have revised this phrase throughout the manuscript. (line 193)
2. Scientific tone and structure
Change the informal expressions to professional tone e.g. face more difficult anti-infection….

**Response**: Thank you. We have changed “face more difficult anti-infection….” to “lead to longer anti-infection treatment”. (line 207)
3. Statistical reporting. Use consistent phrasing for P-values and avoid redundancy.

**Response**: Thanks for your professional advice. We have rewritten the "Statistical" section to use consistent wording for P-values. (line 107-111)
4. Content clarity
1. The connection between biofilm formation and antibiotic resistance could be more directly and clearly explained.

**Response**: We have revised the manuscript to provide a more direct and explicit explanation. (line 245-251)
2. Clarify the contrast between your findings and previous literature where appropriate.

**Response**: Thank you for your professional advice. In the article discussion, we have added our findings and previous comparisons. (line 225-229)
Did you encounter any limitation in this study? If yes, indicate them.

**Response**: We appreciate the reviewer's opportunity to address limitations. Key constraints include: The sample source was relatively single, and there was no data from other medical institutions in the region. There was no description of how *E. coli* enters the urinary system from the digestive system. We've put it all in the revised manuscript. (line 263-272)

**Reviewer #2**:

1. Re-frame the title. Avoid the repeated use of characteristics

**Response**: Thanks for. We have changed the title as “Clinical epidemiological characteristics and antibiotic sensitivity of *Escherichia coli* urinary tract infection”.(line1-2)
2. 2nd paragraph, Line4…” 40% of hospital infections” - do you mean hospital acquired infections? Clarify

**Response**: Thank you for your professional advice. We have changed "hospital infections" to "hospital-acquired infections" in the revised manuscript. (line 56)
3. Same paragraph: The most common gram-negative bacteria is Enterobacteriaceae, mainly E. coli, and Staphylococcus is mainly among Gram-positive bacteria. Replace ‘is’ with are. Uropathogenic Escherichia coli (UPEC) is a group of parenteral pathogenic Escherichia coli should be italicized

**Response**: Thank you for your kind reminder. We have made the changes as your requested. (line 63-64)

4. The subtitle “Experimental procedure” is perhaps more appropriately expressed as laboratory testing

**Response**: Thanks for your suggestion. We have changed "Experimental procedure" to "Sample culture and antibiotic sensitivity testing of isolates" in the revised manuscript. (line 79)
5. Still under the same subtitle…“using the VITEK 2 Compact system of the French company Merrier).” Shorten it like......VITEK 2; Merrier). Also apply to other parts of the manuscript where this occurs.

**Response**: We thank the reviewer for this helpful suggestion to improve conciseness. We have revised all instances of the instrument description according to your recommendation. (line 84-85)
6. Extended-Spectrum β-Lactamases- first use insert (ESBL) after it

**Response**: Thanks for your suggestion. We have inserted “(ESBL) “where it first appeared in the revised manuscript. (line 87)

5. “Hospitalization time” define; explain

**Response**: We revised the manuscript to annotate "Hospitalization time" below Table 1 and standardized its use throughout the text with the following annotation: Hospitalization time refers to the total duration (in days) from hospital admission to discharge for a single clinical episode, excluding time in emergency departments or outpatient clinics prior to formal admission. (line 128-130)

6. “in-hospital infection and out-of-hospital infection.” Define these terms.

**Response**: Thanks for your suggestion. We have changed “in-hospital infection” to “hospital-acquired infection” and “out-of-hospital infection” to “non-hospital acquired infection” in the full manuscript.	The first paragraph in the section “Hospital-acquired infection and Non-hospital acquired infection” is defined and interpreted according to references. (line 132-143)
9. “characteristics of the infected population (including gender, age, hospitalization time, etc.)” Time of the year) not given. This will give temporality to the epidemiological data given

**Response**: Thank you for your reminder. we have revised the manuscript as follows: The clinical data of all cultured *E. coli* patients (including gender, age, hospitalization time, etc.) were collected and analyzed in the whole year of 2023. (line 91-93)

10. Bacterial culture- could be changed to: Sample culture and antibiotic sensitivity testing of isolates

**Response**: Thanks for your suggestion. We have revised “Bacterial culture” to “Sample culture and antibiotic susceptibility testing of isolates” throughout the manuscript to better reflect our workflow. (line 79)

11. “Research on bacterial culture and drug sensitivity test methods” – Delete

**Response**: Thanks for your suggestion. We have removed it.
12. Under subtitle Statistical analysis “This study uses SPSS23.0” uses should be in the past tense. This should be applied across the methods section. English improvement is generally needed.

**Response**: Thanks for your kind reminder. We have rewritten the statistics section and used the past tense grammatically. (line 107-111)
13. Checkout time in Table 1 should be defined

**Response**: Thank you for your kind reminder. We have defined “Checkout time” in section “General situation of research subjects”. (line 123-125)

14. Under the subtitle “In-hospital infection and out-of-hospital infection” the first statement is a repetition. Delete

**Response**: Thanks for your suggestion. We have removed it.
15. Subtitle “A Drug susceptibility of 401 cases of Escherichia coli”: make it simpler. As in: Drug susceptibility of Escherichia coli isolates

**Response**: Thanks for your suggestion. We have changed “A Drug susceptibility of 401 cases of *Escherichia coli*” to “Antimicrobial susceptibility of *E. coli* isolates”. (line 153)
16. May wish to reframe “In contrast, quinolones like levofloxacin (LEV) exhibited the lowest sensitivity rate at 21.9%, warranting heightened attention from clinicians.” As: “Quinolones such as levofloxacin (LEV) exhibited the highest resistance rate to our isolates rate at 78.1% warranting heightened attention from clinicians.” To emphasize the resistance

**Response**: Thank you. It has been revised in the revised manuscript according to your description. (line 165-166)
17. Table 2: Clinical characteristics of 401 patients under different infection conditions. Delete” under different infection conditions”. Compute and add %s in the table

**Response**: Thanks for your suggestions. We have simplified the table title by removing “under different infection conditions” and added percentage values for all categorical variables. (line 152 and Table 2)

18. Table 3: Sensitivity rates of various antibiotics of 401 cases of Escherichia coli. Again, shorten and make simpler. Provide legend below giving the full name of the abbreviations in the table

**Response**: Thanks for your suggestion. We have changed the name of Table 3 to "Susceptibility of 401 *E. coli* isolates to antimicrobial agents" and added a legend below Table 3 to provide full names for all antibiotic abbreviations in the table. (line 172 and 173-177)
19. intra-hospital and out-of-hospital infections – uniformity in terminologies used in essential. Is this the same as “in-hospital infection and out-of-hospital infection” used elsewhere in the text. It’s confusing.

**Response**: Thank you for your reminder. In order to unify the terminology throughout the text, we have replaced "hospital-acquired" with "in-hospital" and "non-hospital acquired" with "out-of-hospital" in the full text of the revised manuscript. (line 145, 146, 149, and 204,etc)
20. “showed significant differences between different infected groups, and the difference was statistically significant detailed data.”. English usage issues

**Response**: Thank you for your reminder. We have revised this sentence to "Statistics showed that there were differences among different infection groups, and the differences were statistically significant". (line 184-186)
21. In the fourth paragraph under discussion, the statement “However, compared with the situation in European countries, the drug resistance rate of Escherichia coli to compound trimethoprim/ sulfamethoxazole has decreased in several European countries (17, 18).” There seems to be no comparison. Perhaps another English challenge.

**Response**: Thank you for highlighting this issue. We have rewritten this part and modified it to "Conversely, in European countries such as Germany, France, and the United Kingdom, the resistance rate of *E. coli* to trimethoprim/sulfamethoxazole in urinary tract infections typically ranged between 10% and 20%. The lowest rate observed was 4.9% in Finland, whereas the highest was 26.7% in Portugal”. (line 226-229)

22. The limitations of the study were not listed or discussed.

**Response**: Thank you. We have added a dedicated "Limitations" subsection to address this. The key limitations include: Lack of mechanism of *E. coli* invaded urinary system, single sample type, short research time span, and lack of multi-center data. (line 263-272)
23. The very last but long last statement requires English editing.

**Response**: Thank you for your reminder. We have made systematic changes to the article.

**Reviewer #3:**

The authors of this paper have identified an opportunity to correlate retrospective hospital case data, samples in a hospital biobank with hospitalization records for urinary tract infection as a result of Escherichia coli. The authors intended approach to associate the antibiotic susceptibility profiles for the E.coli strains and their corresponding Extended-Spectrum β-lactamase status with hospitalization cases and perhaps source of infection is a unique perspective, which if properly articulated can allow hospital systems develop protocols to reviewing antibiotic use or discontinued use within hospital systems.

The authors have however not clearly articulated their thoughts in the current report. The paper will benefit from a major revision across all sections of the paper from abstract to discussion. In the current state, the paper is hard to follow, requiring major editorial input. Some quick but not exhaustive list of highlights below.

There are some vague terminologies used in the paper which, if clearly defined would greatly assist the readers is understanding the flow of thought presented here.

Inconsistencies in the use of word between in-hospital, out of hospital etc>>. This needs to be properly defined and consistent in presentation across all the sections of the paper as it is crucial in the results interpretation. What does out of hospital infection mean? Infections obtained outside the hospital whilst in-hospital refers to infections obtained in the hospital? In this case a strong association can be determined between ESBL status and infection acquisition which would be a relevant finding.

**Response**: Thank you for correcting our unprofessional writing. In the full text, we have replaced "in-hospital" with "hospital-acquired" and "out of hospital" with "non-hospital acquired". (line 26, 27, 29, 91, and146,etc). And the definition of "hospital-acquired" and "non-hospital acquired" in the first paragraph of the "Hospital-acquired infection and Non-hospital acquired infection" section. (line 132-143)

Was bacterial culture on fresh urine samples undertaken in this study or was analysis on retrospective stored bacterial isolates done? Authors are advised to report the precise methods employed in this study.

**Response**: Thank you for pointing out what we are doing. In this study, we first cultivated urine specimens, found samples that cultivated *E. coli*, and collected basic information of the patient, combined with bacterial conditions (whether ESBL is produced and drug sensitivity) and patient basic information to conducted statistical analysis and explore new discoveries. The specific methods were revised in the revised manuscript. (line 80-82, 95-103)

How did the authors arrive at sensitivity values for each antibiotic type? Can this be highlighted under the methods section?

**Response**: Thank you for your question. In the "Sample culture and antibiotic sensitivity testing of isolates" section, we have made modifications and pointed out that all antibiotic susceptibility tests for *E. coli* were conducted using the VITEK 2 system from bioMérieux of France, and all operation processes conformed to the operation norms and procedures. (line 97-99) The VITEK system performs quality control every month and remains in control. (line 100-101）

Under statistical methods, the authors refer to metrological data- please clarify how this relates to the current study!!

**Response**: Thanks for your reminder. We have rewrite the “statistics” section in combination with the statistical methods we have used and the international reporting standards. (line 103-107)

In many cases they repeatedly used Escherichia coli when they could have simply written E.coli.

**Response**: Thanks for your professional advice. We have shortened the word "Escherichia coli" to "*E. col*i" throughout the text.

Also p value is written with the small letter p and not capitalized within text.

**Response**: Thank you for your professional advice. We have written all P values in the text in the lowercase letter p.

ESBL status relative to days of hospitalization was highlighted in table 2 what bearing does this have on the patient statuses i.e. of out-of hospital and in-hospital that come across strongly in this paper. What is the relationship between ESBL and these two definitions? Is that what was intended to be captured in Table 1?

**Response**: Thank you. Table 2 was classified according to the type of infection. Hospital-acquired infection was mainly ESBL-positive bacteria, and the length of hospital stay was longer than that of non-hospital acquired infection, indicating that ESBL-positive patients with hospital-acquired infection are more challenging and need more time when facing antibacterial treatment. Table 1 was a classification according to whether ESBL is produced or not, showed that ESBL-positive patients need longer to face antimicrobial therapy than ESBL-negative patients.

Under the General situation of research subjects in the results section this statement reads ‘A total of 401 study subjects that met the inclusion criteria were included in this study- there was never a place preceding or after this section where the precise inclusion criteria were clearly highlighted, whilst this may be assumed, it would be clearer to the readers if this can be properly defined. Also under the header titled ‘In-hospital infection and out-of-hospital infection’ the statement refers ‘A total of 401 patients were included in this study- should this not rather read as samples from 401 patients or study subjects? Also be consistent for ease of readability. This section will be a good place to further reiterate the definition of in-hospital infection vs out-of-hospital infection. Authors are advised to rewrite the entire section to ensure clarity of message.

**Response**: Thank you for your professional advice. We have made the following modifications based on your suggestions. In the first sentence of the "Sample culture and antibiotic sensitivity testing of isolates" section, we add inclusion specimens (line 76-78) that met this study to clearly indicate to the readers. Under the title "Hospital-acquired infection and non-hospital acquired infection", "A total of 401 patients were included in this study" has been modified to "A total of 401 patient samples were included in this study". (line142) At the same time, in the first paragraph we define hospital-acquired infection and non-hospital acquired infection. (line130-141)
