# Supplementary material for: Clinical epidemiological characteristics and antibiotic sensitivity of Escherichia coli urinary tract infection
Source: PLoS One. 2025 Nov 11;20(11):e0336572. doi: 10.1371/journal.pone.0336572 (PMC12604755; doi:10.1371/journal.pone.0336572)
Supplement: Attachment — Submitted filename: renamed_ff9a6.docx [file pone.0336572.s007.docx]

**Point-by-point response to Referees’ comments**

**Reviewer #1:**

The authors have addressed all the comments; corrected and incorporated the suggested comments and the manuscript reads better and is technically sound. Therefore, I recommend this manuscript.

**Response**:Thank you. Although no additional comments were raised by you during the present revisions, in order to make the readers more readable, we have made changes to the details of the article and improved the manuscript as much as we can. We appreciate your comment on our paper. The revised manuscript has been resubmitted. And hope to consider publishing in *PLOS ONE*.

**Reviewer #2：**

While the term "non-hospital acquired infection" now being used is OK, changing it to the commonly used community acquired infection will make the paper more searchable and visible as well as increase comparability.

**Response**:Thank you for your suggestion, we have replaced all the "non-hospital acquired infection" in the full revised manuscript with "community acquired infection".(line27,29,94,134,etc,table2,4)

Though they did include limitations as requested, the first limitation stated can be deleted: since the apparent aim of the study is to study "the clinical characteristics of Escherichia coli in urinary tract infection and guide empirical treatment," studying only Escherichia coli derived from the urinary tract infection as opposed to from other organs cannot be a limitation. The remaining limitations can be improved for better readability.

**Response**:Thank you for your professional advice, we have removed the first limitation, and the explanation you gave has benefited us a lot. To make readers more readable, we have improved the remaining limitations.(line267-273).

If permitted by the editors, structuring the abstract will give it more clarity and readability especially making clear the aim of the study.

**Response**:Thank you for your proposal. We have modified the abstract section according to the structure of “Introduction, Methods, Results, and Conclusion” according to the editor’s requirements and clarified the purpose of this study at the end of “Introduction”.(line16-18)

**Journal Requirements:**

Please review your reference list to ensure that it is complete and correct. If you have cited papers that have been retracted, please include the rationale for doing so in the manuscript text, or remove these references and replace them with relevant current references. Any changes to the reference list should be mentioned in the rebuttal letter that accompanies your revised manuscript. If you need to cite a retracted article, indicate the article’s retracted status in the References list and also include a citation and full reference for the retraction notice.

**Response**:Thank you for your enthusiastic reminder. We carefully checked the reference list and found that there were indeed inappropriate articles being cited, and we have replaced them based on the content of the article. (References from Articles 7, 11, 27)（line301-304,316-319,375-378）
