# Supplementary material for: Clinical epidemiological characteristics and antibiotic sensitivity of Escherichia coli urinary tract infection
Source: PLoS One. 2025 Nov 11;20(11):e0336572. doi: 10.1371/journal.pone.0336572 (PMC12604755; doi:10.1371/journal.pone.0336572)
Supplement: Attachment — Submitted filename: renamed_d3ee1.docx [file pone.0336572.s008.docx]

**Response to Editor Comments**

Thank you for your thoughtful revisions to the manuscript. Please review the abstract to ensure clarity and smooth flow. Kindly remove the subtitle “Limitations” and incorporate that content into the final paragraph of the Discussion section. Also, check the spelling of *E. coli*—make sure there is a space between “E.” and “coli.” Lastly, please add a period "." to the final sentence of the Discussion section.

**Response**: Thank you for your reminder. We have made slight modifications to the abstract to make the content clearer and the sentences smoother (line17,18, 26-27,31,38-43). Secondly, we have also deleted the subtitle "Limitations" and included it in the last paragraph of the Discussion section (line255-261). We also checked the spelling of all "*E. coli*" in the entire manuscript, and four spellings were found to be inconsistent with the specification (line37, 230,255,258), and a space was added between "E" and "coli"; Finally, a period ". " was added to the last sentence of the original Discussion part(line254). At last, on behalf of our research team, I would like to express my gratitude for your hard work and wish you success in your work and a happy mood.
